# Cervical collar use following anterior cervical hybrid surgery: protocol for a prospective randomized, time-controlled trial

**DOI:** 10.1186/s13063-023-07409-7

**Published:** 2023-06-16

**Authors:** Junbo He, Qingyu Liu, Zijiao Yang, Hao Liu, Tingkui Wu, Chen Ding, Kangkang Huang, Beiyu Wang

**Affiliations:** 1 Department of Orthopedic Surgery, Orthopedic Research Institute, West China Hospital, Sichuan University, No. 37 Guo Xue Rd, Chengdu, 610041 China; 2 West China School of Medicine, Sichuan University, Chengdu, China

## Abstract

**Introduction:**

Cervical hybrid surgery (HS) combines anterior cervical discectomy and fusion (ACDF) and cervical disc arthroplasty (CDA) to establish an individualized surgical plan for patients with multiple cervical disc degenerative diseases. In order to maintain the stability of the spine after HS, an external cervical collar is often used. However, there is still controversy regarding the importance of a cervical collar following surgery. This study aims to determine whether the cervical collar is effective and how long it should be worn after surgery.

**Methods:**

This is a randomized, single-center, prospective, parallel-controlled trial. Eligible participants will be selected according to the inclusion and exclusion criteria. The primary outcome is the neck disability index, which will be evaluated before surgery and at one week, 3 weeks, 6 weeks, 3 months, 6 months, and 12 months following surgery. The secondary outcomes consist of the Japanese Orthopedic Association Scores, MOS 36-item short-form health survey (SF-36), visual analog scale, Pittsburgh Sleep Quality Index (PSQI), Bazaz dysphagia scoring system, Falls Efficacy Scale, cervical collar satisfaction score, neck soft tissue assessment, and Braden Scale, as well as radiologic assessments for cervical lordosis, disc height of the operative levels, fusion rate, range of motion (ROM), and complications including anterior bone loss, prosthesis migration, and heterotopic ossification. The clinical and radiologic examinations were performed by investigators with no therapeutic relationship with the individual patient. All radiographs were examined by one independent radiologist.

**Ethics and dissemination:**

The results of this study will be published in peer-reviewed journals and presented at conferences. Upon completion of this trial, our findings could provide an appropriate cervical collar-wearing guideline for patients receiving HS.

**Trial registration:**

ChiCTR.org.cn ChiCTR2000033002. Registered on 2020–05-17.

## Introduction

Cervical disc degeneration disease (CDDD), including cervical radiculopathy and myelopathy, is a common diagnosis among adult patients and causes significant disability and loss of productivity [1]. Anterior cervical discectomy and fusion (ACDF) was first introduced by Smith, Robinson [2] and Cloward in the 1950s [3] and has been considered the standard treatment for CDDD. ACDF can be utilized to decompress the anterior spinal cord and preserve the stability of the spinal column; however, multilevel ACDF may have a high risk of adjacent segment degeneration (ASD) [4–7]. Cervical disc arthroplasty (CDA) has been shown to be a safe and effective alternative to ACDF. In addition to maintaining physiologic motion, CDA can also restore disc height and some viscoelastic properties, ensure cervical segment mobility, as well as allow earlier return to normal activity [8]. Moreover, the incidence of ASD is significantly lower in patients that underwent ACDF. Nevertheless, the CDA approach is more expensive, and surgical indications are more restrictive [9]. Therefore, cervical hybrid surgery (HS) was proposed by combining ACDF and CDA in treating different levels to provide a better chance for cervical range of motion (ROM) protection and spinal reconstruction [10, 11].

Postoperative collar use provides several advantages, including the restriction of neck flexion, extension, lateral tilt, and rotation [12], and immobilization also reduces pain and provides spinal stability [13, 14], which makes it possible to reduce the risk of complications such as graft subsidence, displacement, and resorption [15–17]. Patients who undergo ACDF are always recommended to wear cervical collars to achieve the results mentioned above, while patients who undergo CDA are theoretically not required to wear collars since the implant prosthesis needs to maintain a physiological range of motion [18]. Collar usage after ACDF has been the subject of various studies in the literature, but there is no consensus on whether collars should be used. Some studies have suggested that collars restrict excess motion and are associated with improved postoperative outcomes [19–21]. In a prospective randomized controlled trial with patients receiving ACDF, the postoperative use of a cervical collar for the first six weeks was associated with a significantly lower neck disability index (NDI) [19]. However, several studies have reported different findings [22–26]. A Randomized clinical trial showed no statistically significant differences between braced and non-braced groups in NDI after 6 weeks post-operation [22, 25]. There was no statistically significant difference in 1-year postoperative Neck Disability Index scores between the brace and no-brace groups according to a prospective randomized control trial for patients who underwent ACDF [23]. Indeed, collar usage was correlated with a higher risk of pressure ulcers [12, 14], swallowing difficulty [27], coughing [28], and even marginal mandibular nerve palsy with long-term sensory degradation [29]. According to the questionnaires assessing the collar patterns of spine surgeons from 2009 to 2021 [30–32], surgeons may continue to use rigid collars primarily due to a lack of quality evidence directly comparing outcomes with or without bracing.

As a result, postoperative cervical collar use remains controversial, demonstrating the necessity of further research into the effects of collar use on clinical and radiographic outcomes after HS. The aim of this prospective, randomized control study was to investigate prospective physical, functional, and quality of life-related outcomes of patients undergoing HS, accompanied by the use of cervical collars for different periods of time.

## Methods

### Study design

This single-center, exploratory, prospective randomized controlled trial will be carried out at the West China Hospital of Sichuan University. Patients who fulfill study entry criteria will be randomly assigned to wear no collar, wear it for 3 weeks, wear it for 6 weeks, and wear it for 12 weeks at a ratio of 1:1:1:1. The study design and protocol were approved by the Ethics Committee on Biomedical Research, West China Hospital of Sichuan University. SPIRIT reporting guidelines were adhered to in this protocol [33].

### Study participants

Eligible participants must be between 18 and 60 years old and will receive continuous double or multilevel cervical hybrid surgery. Patients will be identified and recruited by JH on the day of admission. The study will provide both oral and written information, followed by the acquisition of signed informed consent by a designated spinal surgeon. Patients with systemic metabolic diseases, severe osteoporosis, unstable or abnormal anatomy of the cervical spine or severe stenosis of the cervical spinal canal on imaging, trauma, infection and tumor, and mental illness; participants diagnosed with mental illness or in vulnerable groups, those who underwent surgery for cervical vertebrae, or those who participated in other research projects will be excluded. CDA will be performed at the segment without cervical instability, defined as sagittal plane translation ≤ 3.5 mm and sagittal plane angulation ≤ 20°. The contraindications for CDA include an absence of motion ≥ 2° and facet joint degeneration. ACDF will be chosen if radiographic signs of instability, bridging osteophytes, and facet degeneration are observed.

### Randomization and intervention

**Fig. 1:**
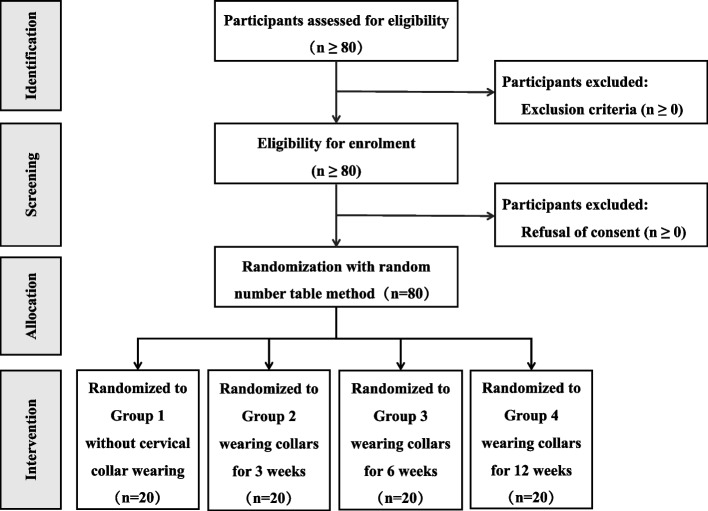
Flow diagram

Upon the establishment and documentation of baseline data, patients shall be allocated into four groups through a random number table generated by QL in the subsequent manner: Group 1 (no cervical collar), Group 2 (cervical collar for 3 weeks), Group 3 (cervical collar for 6 weeks), and Group 4 (cervical collar for 12 weeks). Each group will consist of 20 patients, as shown in Fig. 1. When wearing a cervical collar (Aspen, Vista), it is mandatory for all patients to conform to the suitable height and circumference while maintaining eye level, restricted mobility, and absence of discomfort. It may be necessary to terminate the study if the principal investigator determines there is an unacceptable risk of serious adverse effects.

### Concealment mechanism and implementation

An allocation sequence is enclosed in an envelope with a unique identification number to conceal it. Prior to the point of allocation, both participants and the recruiter (JH) would be unaware of the treatment assignment. The intervention will be assigned by HL, who will be blinded to the randomization file.

### Sample size calculation

The sample size was calculated based on the data results of previous research. The target sample size was 80 (20 in each group). The sample size was calculated assuming NDI means of 10.0 for patients with collars and 5.00 for patients without collars after HS and a standard deviation of 5.00 [23–25], with 5% significance (a = 0. 005) and 90% power (b = 0.90) and considering a dropout rate of 20%. The sample size was calculated using PASS 15 software.

### Blinding

After randomization, patients will not be blinded to whether they will wear a cervical collar. Except for the follow-up personnel, other researchers—including the statisticians, outcome assessors, and data analysts—will all be blinded to the group assignments. The follow-up personnel will not be involved in the outcome assessment or data analysis. The design is open label with outcome assessors, data analysts being blinded, so unblinding will not occur.

### Surgical operation

The same senior spine surgeon will carry out all surgeries. Patients will receive general anesthesia before surgery using the common right-sided anterior cervical approach with their necks in a neutral position. An anterior technique will be used to perform discectomy and decompression at the index level by removing osteophytes, the posterior longitudinal ligament, and disc tissues. A decompression procedure will be initially performed if the degenerative segment is more severe. For CDA, after preparing end plates and disc space with burrs, tests, implant tests, and rail cutter guide. Next, the channels in the end plates and a Prestige LP disc of the proper size will be installed (Medtronic Sofamor Danek, Memphis, TN, USA). For the ACDF approach, the intervertebral space will be filled using Zero-P (Synthes, Oberdorf, Switzerland) implants packed with tricalcium phosphate or locally removed bone. All prostheses will be implanted with the aid of fluoroscopy.

### Outcomes

#### Primary outcome

We chose the Neck Disability Index (NDI) functional score as the primary study endpoint. The NDI was proven to have better reliability, validity, and responsiveness for self-rated disability for postoperative patients [34]. The NDI will be evaluated postoperatively and at the 1^st^ week, 3^rd^ week, 6^th^ week, 3^rd^ month, 6^th^ month, and 12^th^ month after surgery. The NDI consists of ten items, including pain intensity, personal care, lifting, sleep, driving, recreation, headaches, concentration, reading, and work. Each item is scored out of 5 for a maximum total score of 50. Neck functions will be assessed by this score for all patients [35].

#### Secondary outcomes

**Fig. 2:**
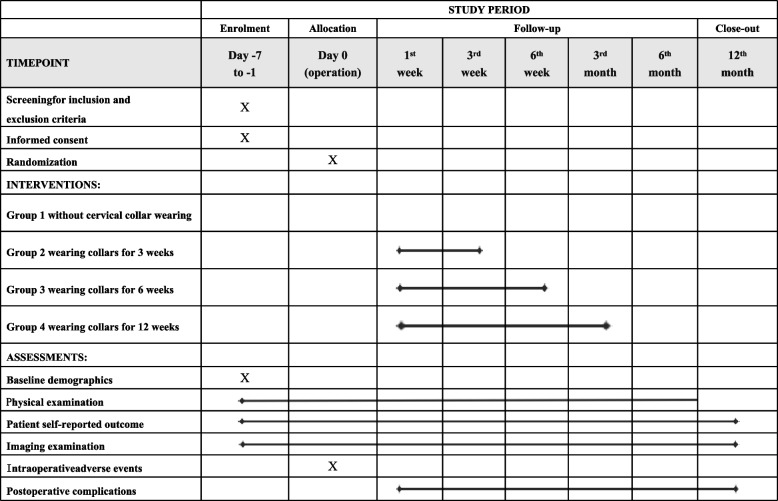
SPIRIT figure of this trial

The secondary outcomes will include clinical, radiologic, and complication assessments. The assessment will be conducted prior to the operation and subsequently after the operation, continuing up to a period of 12 months post-operation or until the conclusion of the study (Table 1 and Fig. 2). The clinical and radiologic examinations will be performed by investigators who have no therapeutic relationship with the individual patient. All radiographs will be examined independently by one radiologist.

**Table 1:** Data collection

Variables	Baseline	Follow-ups					
	Preoperation	1^st^ week	3^rd^ week	6^th^ week	3^rd^ month	6^th^ month	12^th^ month
Screening for inclusion and exclusion criteria	√						
Informed consent	√						
Randomization	√						
Baseline demographics	√						
Information collected through telephone follow-up/subjective scales							
NDI	√	√	√	√	√	√	√
JOA	√	√	√	√	√	√	√
VAS	√	√	√	√	√	√	√
PSQI	√	√	√	√	√	√	√
BDS	√	√	√	√	√	√	√
FES	√	√	√	√	√	√	√
Information collected through questionnaire follow-up/subjective scales							
SF-36	√	√	√	√	√	√	√
Cervical collar satisfaction score		√	√	√	√	√	√
Information collected through physical examination/objective scales							
Neck soft tissue assessment	√	√	√	√	√	√	√
Braden Scale		√	√	√	√	√	√
Information collected through radiologic examinations							
Cervical lordosis	√	√		√	√	√	√
ROM of C2-C7	√	√		√	√	√	√
Disc angle of the operative levels	√	√		√	√	√	√
ROM of the operative levels	√	√		√	√	√	√
ROM of the adjacent levels	√	√		√	√	√	√
Disc height of the operative levels	√	√		√	√	√	√
Fusion rate		√		√	√	√	√
Complications							
Anterior bone loss		√	√	√	√	√	√
Prosthesis migration		√	√	√	√	√	√
Heterotopic ossification		√	√	√	√	√	√

*JOA* Japanese Orthopedic Association, *NDI* Neck Disability Index, *VAS* visual analog scale, *PSQI* Pittsburgh Sleep Quality Index, *BDS* Bazaz dysphagia scoring system, *FES* Falls Efficacy Scale, *SF-36* MOS 36-item short-form health survey, *ROM* range of motion

### Clinical examinations

#### Subjective scales

1. JOA: The JOA score is used to evaluate the neurological status of patients with myelopathy. The JOA of the cervix has 4 main parts: upper and lower limb motor function and sensory and bladder function [35, 36].
2. SF-36: In addition to the JOA, SF-36 is intended to serve as a general health status indicator. The validity and reliability of the SF-36 have also been established in patients with cervical myelopathy [37].
3. VAS: VAS scores will be used to assess neck and upper limb pain in all patients [38].
4. Pittsburgh Sleep Quality Index, PSQI: The PSQI is considered an accepted reference or gold standard for self-perceived sleep quality [39].
5. The Bazaz dysphagia scoring system.
6. Falls Efficacy Scale: It is designed to measure self-perceived fear of falling during l4 common activities [40].
7. Cervical collar satisfaction score: It is designed to evaluate whether participants are content with cervical collars use.

#### Objective scales

8. Neck soft tissue assessment and the Braden Scale will be used to evaluate Neck soft tissue injury extent within 10 cm around the collar position of patients in the collar groups [41] (Table 1 and Fig. 2).

#### Radiologic examinations

**Fig. 3:**
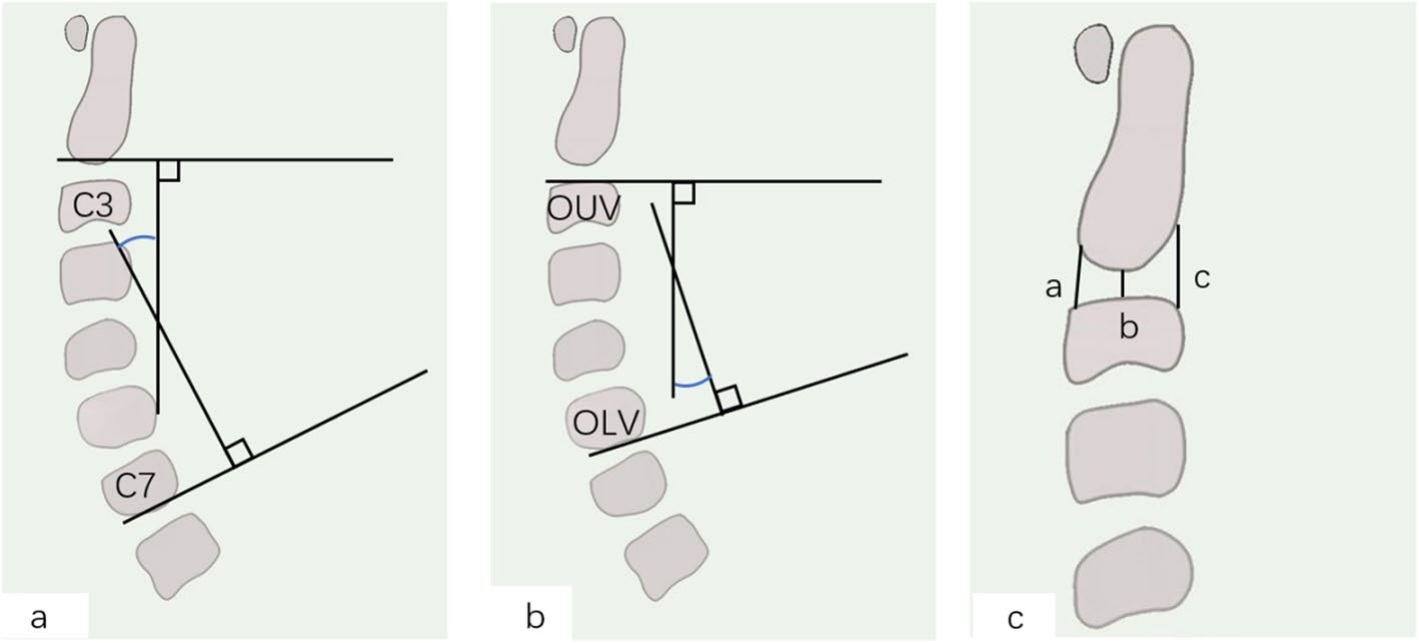
Measurement of cervical lordosis, disc angle of the operative levels, ROM, and disc height of the operative levels. 1**a** Angle of C2-C7 Cobb includes drawing a line either parallel to the inferior endplate of C2 or another line parallel to the inferior endplate of C7. Perpendicular lines are then drawn from each of the 2 lines noted above, and the angle subtended between the crossing of the perpendicular lines is the cervical curvature angle. 1**b** A line was drawn either parallel to the superior endplate of the operative upper vertebra (OUV) or another line was drawn parallel to the inferior endplate of the operative lower vertebra body (OLV). Perpendicular lines are then drawn from each of the 2 lines noted above, and the angle subtended between the crossing of the perpendicular lines is the cervical curvature angle. The ROM is defined as the difference in Cobb angle between flexion and extension in lateral radiographs. 1**c** The intervertebral space height equals one-third of the sum of the anterior intervertebral space height (a), middle intervertebral space height (b), and posterior intervertebral space height (c). Intervertebral space height = (a + b + c)/3

9. Cervical lordosis: It was defined as the angle between the inferior end plate of C2 and the inferior end plate of C7. Patients need to take radiographs of the cervical spine function position.
10. Disc height of the operative levels: Intervertebral space height equals one-third of the sum of anterior intervertebral space height, middle intervertebral space height and posterior intervertebral space height (Fig. 3)
11. Cobb angle and ROM (Table 2 and Fig. 3) [42, 43].
12. Bony fusion: Bony fusion is defined as continuous trabecular bone formation on cervical vertebrae reconstruction CT, cervical intervertebral range of motion of full extension and flexion of less than 2° and radiolucency covering the implant’s outer surfaces of less than 50%. All criteria must be met for a joint to be considered effectively fused [44, 45].

**Table 2:** The measurement method of Cobb angle and ROM

Items	Definitions
Cobb angle	The angle between the lower endplate of the upper vertebral body and the upper endplate of the lower vertebral body
ROM	The difference in Cobb angle between full flexion and extension in lateral radiographs
ROM of the operative levels	The difference in Cobb angle of the operative levels between full flexion and extension in lateral radiographs
ROM of the adjacent levels	ROM of the adjacent (upper and lower) levels of operative levels

*ROM* range of motion

#### Complications

13. Anterior bone loss: It was as a combined standard of the percentage of the endplate length and implant subsidence [44] (Table 1).
14. Prosthesis migration: It was defined as the sum of the change in the height of the cranial and caudal vertebral body between the immediate postoperative and follow-up situations on lateral radiographs. A level was classified as subsided if the measured subsidence was > 2 mm. Anteroposterior migration is defined as the sum of the cranial and caudal translation of the prosthesis with respect to the corresponding endplates between the immediate postoperative and follow-up situations on lateral radiographs. A prosthesis was classified as migrated if the anteroposterior migration was > 3 mm [45].
15. Heterotopic ossification (HO): HO at the index level will be evaluated using the scoring system established by Mehren et al. [46] (Table 1).

### Data collection

To ensure a satisfactory follow-up rate, we chose telephone follow-up to evaluate the NDI, JOA, VAS, Pittsburgh sleep quality index, the Bazaz dysphagia scoring system, Falls Efficacy Scale, and cervical collar satisfaction score. Since responding to many items is time-consuming for patients, the SF-36 health survey will be collected through questionnaires. When collecting data, the repeated parts will be asked only once to lower the confusion and improve the follow-up rate. Complications and cervical imaging parameters need to be evaluated through radiographs and CT. For a higher follow-up rate, healthcare professionals are to be reminded regularly (Table 1 and Fig. 2).

### Follow-up time

Clinical data were collected preoperatively and at routine postoperative intervals of 1, 3, 6 weeks, 3, 6, and 12 months, as well as at the last follow-up visit. Radiographs and CT scans were routinely taken preoperatively and at postoperative intervals of 1, 3, and 6 weeks and 3, 6, and 12 months as well as at the last follow-up period.

### Oversight and supervision

The study is under the supervision of the research team, comprising orthopedic surgeons, radiologists, data analysts, and other pertinent staff. In terms of research quality control and quality assurance, the primary research team members will convene on a monthly basis to ensure the smooth operation of the trial.

### Data monitoring

An independent committee will monitor data annually. Any event that has a reasonable, causal relationship to the study intervention, including pressure ulcers, mild swallowing difficulty, coughing, muscle stiffness, nerve palsy, pseudarthrosis, and vertebral body collapse, will be deemed an adverse event and promptly reported to investigators for evaluation. In accordance with the stipulations pertaining to anticipated, severe, and causal occurrences, adverse events are thoroughly recorded, fully processed, tracked, and reported to the Ethics Committee promptly until properly resolved or stable. The principal investigator will conduct a cumulative review of all adverse events once a quarter and convenes an investigator meeting to assess the risks and benefits of the study when necessary. No formal audit is scheduled for this trial.

### Statistical analysis

We used SPSS version 20.0 (IBM Corp, Armonk, NY, USA) for standard statistical analyses. Quantitative variables are presented as the mean ± standard deviation when normality is met. We used one-way analysis of variance (ANOVA) to compare quantitative data. If *p* < 0.5 for ANOVA, LSD or Dunnett’s test was used to compare means. Chi-square analysis or Fisher’s exact test was used to compare qualitative data, and paired sample *t*-tests were used for the same group. Comparisons of unidirectional orderly data were analyzed using the Wilcoxon signed-rank test. Two-sided *P* values were reported when comparing differences between the 2 groups. *P* value < 0.05 was considered of statistical significance.

### Dissemination plan

The trials results will be communicated through the presentation at academic conferences and publication in peer-reviewed medical literature.

## Discussion

External cervical brace fixation is often performed after anterior cervical surgery to provide biomechanical support to maintain spinal physiological curvature and stability, avoiding complications such as laryngeal edema and hematoma [47, 48]. There is still some debate regarding whether cervical collars should be used following surgery [24]. Previous studies have shown that the use of a cervical collar can reduce the risk of graft nonunion, graft displacement and sinking. At the same time, it can also restrict neck movement, providing spinal stability, reducing pain, and even increasing the sense of security in patients [20, 49]. Despite these benefits, some researchers have contested their validity. In their study, they found that patients with cervical pain that did not wear a cervical collar did not have a lower VAS score than those who wore a collar for 2 weeks. Moreover, the JOA score, SF-36, ROM, anterior convex angle, and complications were comparable between these two groups [50, 51]. In addition, wearing cervical collars can also bring many complications to patients, such as skin injuries skin ulcers, impaired daily activities and sleep, and decreased lung capacity and function [52–54].

Because there are no scientific clinical trials verifying the efficacy of cervical collars after hybrid surgery [20, 24, 26, 55–57], to explore the effect of wearing neck braces after undergoing hybrid surgery and discuss the best wearing time for recovery, we designed this randomized controlled trial, hoping to gain scientific and accurate results about the physical, functional, and quality of life-related outcomes of patients.

The process of osseointegration involves the direct structural and functional connection between living bone and the implant. This is normally initiated 6 weeks postoperatively, while the bone-graft bone bridge forms in around 3-month time. Therefore, most complications, including graft subsidence and loss of cervical lordosis appear during the first 6 weeks following surgery [58–60]. Thus, 3 months post-surgery is a critical time for recovery. In that case, the collar immobilization times were all set within 3 months post-surgery. Then, we adjusted the wearing time of the cervical collar to 3 weeks, 6 weeks, and 12 weeks.

Previous studies have merely selected NDI, SF-36, bone fusion rate, and imaging methods to observe the results of cervical collar wearing [20, 23–25]. In addition to selecting NDI as the primary outcome, we also designed detailed secondary indicators to monitor the possible complications caused by collar use. For instance, the JOA and the Bazaz dysphagia scoring system scoring system will be used to assess the impact of cervical collars on patients' daily life, while patients’ sleep quality will be monitored using the PSQI. Moreover, the cervical collar satisfaction score is designed to evaluate the feelings of patients with cervical collars from a subjective perspective.

Compared with previous studies [20, 23–25], we have added more details since our follow-up time may be extended to up to one year. Moreover, we used a variety of methods to evaluate the effectiveness of neck braces. Since patients often cannot return for reassessments, we opted to use phone calls for follow-ups. Radiographs and CT scans are necessary to evaluate complications and cervical imaging parameters; thus, we would allow such patients to have it done at their local hospitals. To guarantee a rigorous scientific study, patients will be screened strictly in accordance with the inclusion and exclusion criteria. Next, physicians will teach each patient how to correctly wear their neck braces to avoid unnecessary impacts on the ultimate findings. Finally, for patients who are not wearing neck braces, it is imperative that we explain the reasons in detail to reduce the placebo effect.

Nevertheless, there are several limitations to our study design. First, our trial is a single-center study; therefore, generalizing our findings to other centers should be done cautiously. Second, patients cannot be blinded in our study since the intervention is evident.

In summary, our study aims to utilize a rigorous design to comprehensively explore the effectiveness of cervical collar use by assessing various outcome measures following hybrid surgery. As a result of this trial, the feasibility of the protocol will be assessed, and a postoperative recovery strategy that is more considerate of patients and minimizes complications will be developed.

## Trial status

Recruitment began in June of 2020. Recruitment is expected to be complete by June 2023. The current Protocol version is 2.0, dated 4/1/20.

## Data Availability

The datasets analyzed during the current study and statistical code are available from the corresponding author on reasonable request, as is the full protocol.
